# Risk factors for mortality and effect of correct fluid prescription in children with diarrhoea and dehydration without severe acute malnutrition admitted to Kenyan hospitals: an observational, association study

**DOI:** 10.1016/S2352-4642(18)30130-5

**Published:** 2018-07

**Authors:** Samuel Akech, Philip Ayieko, David Gathara, Ambrose Agweyu, Grace Irimu, Kasia Stepniewska, Mike English, Clinical Information Network authors, Samuel Ngarngar, Nick Aduro, Loice Mutai, David Kimutai, Caren Emadau, Cecilia Mutiso, Celia Muturi, Charles Nzioki, Francis Kanyingi, Agnes Mithamo, Magdalene Kuria, Samuel Otido, Anne Kamunya, Alice Kariuki, Peris Njiiri, Rachel Inginia, Melab Musabi, Barnabas Kigen, Grace Akech Ochieng, Lydia Thuranira, Morris Ogero, Thomas Julius, Boniface Makone, Mercy Chepkirui, James Wafula

**Affiliations:** a Kenya Medical Research Institute/Wellcome Trust Research Programme, Nairobi, Kenya; b Department of Paediatrics and Child Health, University of Nairobi, Nairobi Kenya; c Centre for Tropical Medicine, Nuffield Department of Clinical Medicine, University of Oxford, Oxford, UK; d Worldwide Antimalarial Resistance Network, Oxford, UK

## Abstract

**Background:**

Diarrhoea causes many deaths in children younger than 5 years and identification of risk factors for death is considered a global priority. The effectiveness of currently recommended fluid management for dehydration in routine settings has also not been examined.

**Methods:**

For this observational, association study, we analysed prospective clinical data on admission, immediate treatment, and discharge of children age 1–59 months with diarrhoea and dehydration, which were routinely collected from 13 Kenyan hospitals. We analysed participants with full datasets using multivariable mixed-effects logistic regression to assess risk factors for in-hospital death and effect of correct rehydration on early mortality (within 2 days).

**Findings:**

Between Oct 1, 2013, and Dec 1, 2016, 8562 children with diarrhoea and dehydration were admitted to hospital and eligible for inclusion in this analysis. Overall mortality was 9% (759 of 8562 participants) and case fatality was directly correlated with severity. Most children (7184 [84%] of 8562) with diarrhoea and dehydration had at least one additional diagnosis (comorbidity). Age of 12 months or younger (adjusted odds ratio [AOR] 1·71, 95% CI 1·42–2·06), female sex (1·41, 1·19–1·66), diarrhoea duration of more than 14 days (2·10, 1·42–3·12), abnormal respiratory signs (3·62, 2·95–4·44), abnormal circulatory signs (2·29, 1·89–2·77), pallor (2·15, 1·76–2·62), use of intravenous fluid (proxy for severity; 1·68, 1·41–2·00), and abnormal neurological signs (3·07, 2·54–3·70) were independently associated with in-hospital mortality across hospitals. Signs of dehydration alone were not associated with in-hospital deaths (AOR 1·08, 0·87–1·35). Correct fluid prescription significantly reduced the risk of early mortality (within 2 days) in all subgroups: abnormal respiratory signs (AOR 1·23, 0·68–2·24), abnormal circulatory signs (0·95, 0·53–1·73), pallor (1·70, 0·95–3·02), dehydration signs only (1·50, 0·79–2·88), and abnormal neurological signs (0·86, 0·51–1·48).

**Interpretation:**

Children at risk of in-hospital death are those with complex presentations rather than uncomplicated dehydration, and the prescription of recommended rehydration guidelines reduces risk of death. Strategies to optimise the delivery of recommended guidance should be accompanied by studies on the management of dehydration in children with comorbidities, the vulnerability of young girls, and the delivery of immediate care.

**Funding:**

The Wellcome Trust.

## Introduction

Mortality from diarrhoea has decreased since the 1990s, but it still causes up to 0·6 million deaths annually in children younger than 5 years, with most deaths occurring in Africa and southeast Asia.^1^, ^2^ A range of interventions might have contributed to this reduction, such as safe water and hygiene, handwashing, exclusive breastfeeding, measles vaccine, improvement in use of oral rehydration salts,^3^ and zinc supplementation.^4^ Routine rotavirus vaccination is expected to further reduce diarrhoeal severity, morbidity, and mortality, although recent studies have shown that bacterial pathogens, such as non-typhoidal *Salmonella, Cryptosporidium, Shigella*, and *Escherichia coli*, are important causes of diarrhoeal deaths within hospitals.^5^ As a result, there is a global call to prioritise examination of risk factors for continued diarrhoeal mortality and investigate delivery of proven interventions.^6^

Understanding the presenting clinical features that identify children at risk of death is important for front-line clinicians who often solely rely on clinical signs to prioritise immediate care. Standardised WHO guidance^7^, ^8^ on management of diarrhoea with dehydration, which recommends fluid treatment, is available and its usage can modify the association between certain clinical risk factors and mortality. Therefore, examination of risk factors for mortality should also investigate any moderating effect of treatment. We investigate clinical risk factors for in-hospital death and risk modification associated with intended use of WHO fluid treatment guidance in children with diarrhoea and dehydration admitted to 13 hospitals in Kenya.^9^ We use a large routine dataset, based on documentation of clinical practice outside specific research settings, with variation likely in compliance with clinical guidance and contextual factors (captured at the hospital level).

**Research in context**

**Evidence before this study**

Diarrhoea still causes up to 0·6 million deaths annually in children younger than 5 years. Research into the optimisation of delivery and scaling up of existing interventions are thought to be the most urgent priorities to further reduce mortality. However, identification of risk factors for diarrhoeal deaths is also among the top ten research priorities for the reduction of diarrhoeal mortality. To identify studies of risk factors for mortality from diarrhoea in children (younger than 18 years), we searched PubMed for studies published in English between database inception and June 31, 2017. We used various combinations of the following search terms: “diarrhoea”, “diarrhea”, “risk”, “odds”, “mortality”, and “death”. We also perused the bibliographies of retrieved articles. We found case-control studies that reported on risk factors of diarrhoeal deaths in children in resource-poor settings but found none that used routine clinical data. No studies have investigated the effect of use of recommended rehydration guidance on mortality in routine clinical practice. Most available studies have been done in the context of investigating microbial aetiology of diarrhoea rather than clinical characteristics, which are what front-line clinicians use for making treatment decisions in resource-poor settings.

**Added value of this study**

This study shows that that children with other signs of severe illness (suggestive of comorbidity), and not signs of dehydration alone, are most at risk of in-hospital death from diarrhoea and dehydration. Our findings also show that comorbidities are common and that correct fluid prescription is associated with reduced risk of death in these patients.

**Implications of all the available evidence**

These findings highlight the need for further studies on how to manage diarrhoea and dehydration complicated with comorbidities. This research is especially important given that different fluid management approaches could be recommended for children with various comorbidities. We also show the benefit of adherence to recommended practice at the hospital level. This study also raises questions regarding the need to investigate appropriate strategies to optimise delivery of recommended guidance in routine settings in hospitals within resource-poor settings, which often have inadequately trained clinical personnel.

## Methods

### Study design and participants

For this observational, association study, we analysed prospective routine clinical information on admission, immediate treatment, and discharge, which was collected from 13 first referral-level Kenyan hospitals that constitute the Clinical Information Network (CIN). We excluded one CIN facility from this analysis because it is contextually different from the other CIN facilities; it is staffed by non-physician clinicians (ie, non-degree trained clinicians) and is a health centre with inpatient beds rather than being a first-referral hospital. A detailed description of CIN facilities has been previously published.

We screened the database for children meeting the following criteria: age 1–59 months (as guidelines only apply to this group), diarrhoea as a presenting symptom, and a full dataset available. We excluded children with only a minimal dataset or severe acute malnutrition because children with severe acute malnutrition have different fluid treatment guidelines.^8^ Then we identified children with diarrhoea as a presenting symptom or diagnosis or with dehydration as a diagnosis from the full dataset. Within this group, we included in our analyses only children with both diarrhoea as a presenting complaint or diagnosis and also a primary or secondary diagnosis of dehydration (some, severe, shock, or unclassified). These criteria defined a population eligible for treatment according to WHO and Kenyan guidelines for diarrhoea and dehydration. Diarrhoea was considered only a presenting symptom rather than a diagnosis in children classified as having no dehydration on the standard admission form. Data for HIV status were not recorded comprehensively in this population, but the analysis included children with a diagnosis of HIV. Maternal HIV prevalence in Kenya is 6% and the cumulative 5 year mother-to-child transmission rate is 15%; as such, we expect only a small proportion of children in the dataset to have undiagnosed HIV infection.^10^

The Kenya Medical Research Institute (KEMRI) Scientific and Ethical Review Committee approved the CIN study enabling use of de-identified data without individual patient consent.

### Procedures

The hospitals use WHO and locally adapted guidance for management of common conditions.^8^, ^9^, ^10^, ^11^ In brief, these hospitals have implemented two clinical data collection tools (standard paediatric admission records and discharge forms) and have a dedicated data clerk who enters information about admission, treatment, and discharge, once the patient is discharged, into a non-proprietary electronic tool.^12^, ^13^ The clerks are trained and regularly updated on how to abstract data from medical notes and treatment sheets, including fluid prescription sheets, and on how to interpret them. Error checks are done before the data are uploaded and synchronised into a central server, in which further quality checks are done. Any discrepancies noted at this stage are raised with respective clerks who reconcile them. Periodic visits are made to the participating hospitals by the data management team who re-enter a number of randomly selected files to ascertain the accuracy of data entered by the clerks. A minimal dataset, which consists of data required for the routine health information system from all admissions (patient age, sex, diagnoses, and outcome), is collected for a random sample of otherwise eligible admissions in two high-volume hospitals (to reduce the data entry workload) and in all 13 hospitals for surgical or burns cases, admissions younger than 1 month, and admissions during periods when the single clerk is on leave. The randomisation sequence is system generated automatically and not within the clerks' control. A full dataset on clinical presentation, diagnoses, treatments, and outcomes is collected on all other cases and at all other times. We aimed to investigate clinical signs associated with mortality and whether prescription of recommended fluid guidance is associated with a reduced risk of mortality.

### Outcomes

We aimed to examine clinical risk factors for in-hospital death and risk modification associated with intended use of WHO fluid treatment guidance in children admitted with diarrhoea and dehydration across the hospitals.

### Data analysis

Patient characteristics examined include sex, age (≤12 months or >12 months), duration of diarrhoea (≤14 days or >14 days), length of illness (≤2 days or >2 days), history of bloody diarrhoea, malaria status, and abnormal signs obtained on examination of various systems organised as airway, breathing (respiratory system), circulation, hydration status, and disability (neurological system). A child was deemed to have an abnormal system if any sign within the specific system was abnormal (see panel 1 for definitions of abnormal signs). In the case of dehydration, we also created a variable to represent children with clinical signs indicative of severe dehydration (defined in Kenyan guidelines as the presence of both sunken eyes and delayed skin pinch). Correct fluid prescription was defined based on WHO and Kenyan guidance (panel 2).

Panel 1 **Definition of terms**

**Airway signs**

Abnormal airway signs* (only stridor analysed this study) .

**Circulatory signs**

Presence of any one or more of the following: capillary refill time greater than 2 s (delayed capillary refill time), temperature gradient (cold hands and feet), weak pulse volume, or pallor.

**Comorbidity**

Dehydration plus any of the following: malaria, pneumonia, HIV, tuberculosis, anaemia, meningitis, rickets, or asthma.

**Dehydration signs**

Presence of either delayed skin pinch (greater than 1 s) or sunken eyes, or both.

**Impaired circulation**

Presence of any one or more of the following: weak pulse volume, temperature gradient (skin temperature up to shoulder or elbow), or capillary refill time longer than 2 s.

**Impaired consciousness**

AVPU (Alert, Voice, Pain, Unresponsive) score less than A.

**Malaria endemic zone**

A hospital was regarded as located in a high malaria endemic zone if malaria diagnoses comprised more than 50% of admission diagnoses.

**Neurological or disability signs**

Presence of any one or more of the following: convulsions, neck stiffness, bulging anterior fontanelle, inability to drink or breastfeed, or impaired consciousness.

**Pneumonia**

History of cough or difficulty breathing, age older than 60 days, lower chest wall indrawing or tachypnoea-non-severe pneumonia, and any danger sign (oxygen saturation <90%, cyanosis, inability to drink or breastfeed, impaired consciousness, or grunting).

**Respiratory illness signs**

Presence of any one or more of grunting, tachypnoea, chest indrawing, acidotic breathing, crackles, or crepitations.

**Severe acute malnutrition**

Defined as clinical diagnosis of severe acute malnutrition, mid-upper arm circumference less than 11·5 cm, or weight for height *Z* score less than −3 standard deviations.

**Clinical shock**

Clinical diagnosis of shock made by clinician or fluid bolus given.

**Tachypnoea**

Respiratory rate more than 50 breaths per min if aged 12 months or younger, or more than 40 breaths per min if older than 12 months.

**WHO shock**

Presence of all of an AVPU score less than A, weak pulse, and capillary refill time longer than 3 s in the presence of diagnosis of dehydration.

Panel 2 **Definitions of correct fluid prescription based on WHO and Kenyan guidance**

In this study, either WHO plan B, WHO plan C, or shock management^7^, ^8^ were correctly prescribed.

Plan B was correct when given to children classified as having some dehydration and who had not been prescribed bolus fluid and received oral fluid (prescribed for a duration of 4 h or prescribed to be given at regular intervals) or prescribed intravenous fluid (not plan C or fluid bolus and volume <200 mL/kg for a duration of 24 h) plus oral fluid.

Plan C was correct when given to children with a diagnosis of severe dehydration, who had not been prescribed bolus, and in whom oral fluid was used. Correct volume of plan C was 30 mL/kg for step 1 and 100 mL/kg for step 2. The correct duration of plan C was 6 h or less.

Shock management was correct in children indicated as having shock and prescribed fluid bolus plus correct plan C. Intravenous fluid or bolus was correct if normal (0·9%) saline or Ringer's lactate was used.

We did multiple imputation using chained equations to deal with missing data. Using Stata version 15.1, we did imputation with 100 iterations to produce ten imputed datasets on the assumption that co-variable data were missing at random.^14^, ^15^ The imputation model included all variables to be considered as risk factors, auxiliary variables (fever, history of vomiting, and cough), and outcomes, but we excluded any variable with greater than 30% missingness.^16^ We studied risk factors for overall in-hospital mortality and early (within 2 days) in-hospital mortality using mixed-effects logistic regression models, with patient-level data (level I) nested within hospitals (level II) and hospital location in malaria zone as a level II fixed effect. Univariable models (unadjusted) were fitted on the imputed datasets for each patient characteristic and malaria zone location as fixed effects, and hospital intercept as a random effect. The multivariable model (model II; adjusted) was constructed using all patient characteristics and a backward variable selection procedure with a p value for exclusion of 0·05, while maintaining the same multilevel structure as the univariable model. A priori, we decided to include age, sex, and malaria diagnosis in the adjusted model. Final model estimates were derived using Rubin rules.^14^ Final risk factors are variables independently associated with outcome in the multivariable model (Wald test p value <0·05). Because our analysis focuses on a population in whom we have excluded no dehydration, we also investigated the association between signs identified as risk factors in model II and mortality in a broader population of children admitted with diarrhoea using the same approach as done for those with diarrhoea and dehydration to investigate the generalisability of findings.

We investigated effect modification of admission fluid prescription on risk of death in children with signs of dehydration, abnormal respiratory signs, impaired circulation, anaemia, and abnormal neurological signs, by including a binary term for correct initial fluid prescription in the model for early death (within 2 days from admission). We calculated the relative excess odds due to interaction, the attributable proportion, and the multiplicative interaction odds ratio (OR).^17^ Interactions were analysed in the final model (model II) one at a time. Analysis for effect modification was restricted to early deaths because we hypothesised that this is the group whose outcome might be affected by fluid management at admission. Our database collected only fluid prescribed at admission. Patients with no information on fluid management were excluded from the analysis for effect modification.

### Data sharing statement

Data for this report are under the primary jurisdiction of the Ministry of Health in Kenya. Enquiries about using the data can be made to the KEMRI-Wellcome Trust Research Programme Data Governance Committee.

### Role of the funding source

The funders of the study had no role in study design, data collection, data analysis, data interpretation, or writing of this manuscript. SA, PA, DG, AA, GI, KS, and ME had access to the raw data. The corresponding author had full access to all the data in the study and had final responsibility for the decision to submit for publication.

## Results

**Figure 1:**
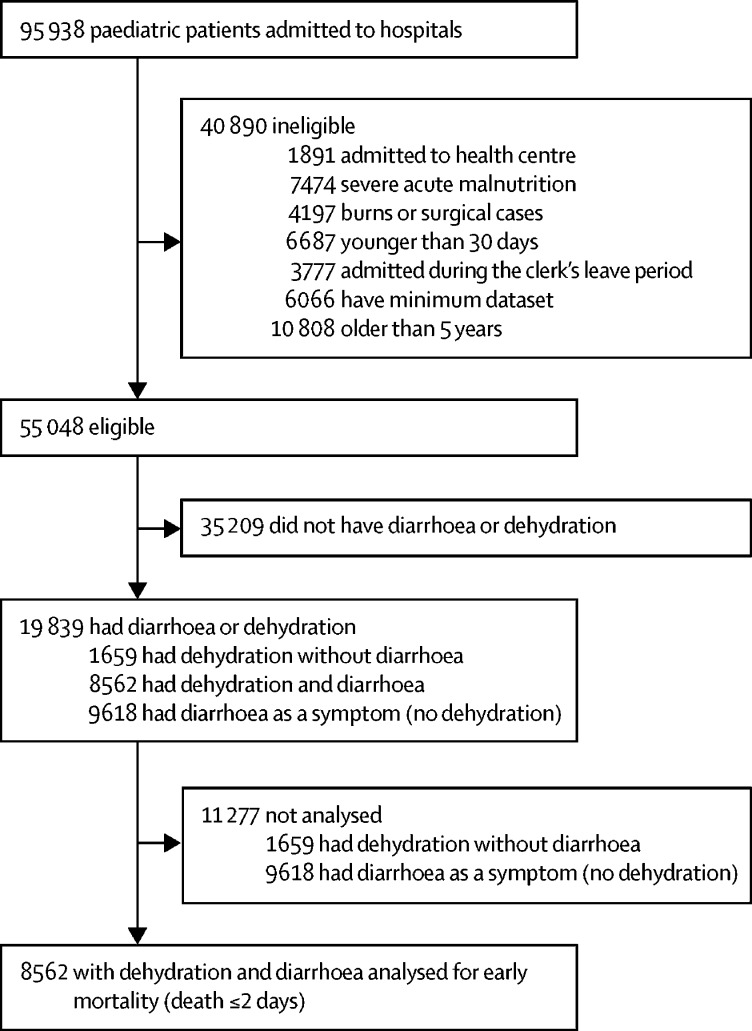
Study profile CIN=Clinical Information Network.

Between Oct 1, 2013, and Dec 1, 2016, 19 839 (36%) of 55 048 eligible paediatric patients with a full dataset admitted to the 13 CIN hospitals had diarrhoea or dehydration. 8562 patients had both diarrhoea and dehydration and 9618 had diarrhoea as a symptom only (without dehydration; figure 1). We did the analysis to determine risk factors and effect modification with fluid therapy in children who had both diarrhoea and dehydration (n=8562).

Among those with diarrhoea and dehydration (n=8562), 5160 (60%) had some dehydration, 2434 (28%) had severe dehydration, 431 (5%) had shock, and 537 (6%) had no classification for the degree of dehydration (table 1). The overall mortality was 9% (759 of 8562) and diarrhoea and dehydration together were associated with 28% (759 of 2711) of all deaths in eligible children in the full dataset. Case fatality in children with some dehydration was 5% (240 of 5160), severe dehydration was 13% (310 of 2434), shock was 42% (179 of 431), and in unclassified cases was 6% (30 of 537). 205 (27%) of the 759 deaths occurred within the first day and a total of 486 (64%) had died within 2 days; median duration to death was 1 day (IQR 0–2). At baseline, fever (6153 [76%] of 8067) and vomiting (6780 [83%] of 8169) were common. Although only 37 (<1%) of 8562 patients fulfilled the WHO criteria for shock (panel 1), a clinical diagnosis of shock was present in 537 (6%) of 8562 patients. Most participants (7184 [84%] of 8562) had a second diagnosis (comorbidity), and these included, among others, slide-positive malaria (2766 [32%]), pneumonia (3065 [36%]), anaemia (428 [5%]), and possible meningitis (642 [8%]). Median ages for admitted patients was 12·0 months (IQR 8·0–18·0).

**Table 1:** Clinical characteristics at admission of patients with diarrhoea and dehydration

		**Complete dataset**		**Missing data for variable (n=8562)**	**Mean proportion with characteristic present in imputed data (n= 85 620)**
		Total	Characteristic present		
Demographics					
	Girls	8485	3724 (47%)	77 (1%)	37 574 (44%)
	Age ≤12 months	8562	4941 (58%)	0	49 410 (58%)
History					
	Length of illness >2 days	8104	5353 (66%)	458 (5%)	56 292 (66%)
	Diarrhoea >14 days	7465	252 (3%)	1097 (13%)	2843 (3%)
	Diarrhoea bloody	7585	277 (4%)	977 (11%)	3174 (4%)
Airway					
	Stridor	7453	97 (1%)	1109 (13%)	1147 (1%)
	Airway signs	7453	97 (1%)	1109 (13%)	1147 (1%)
Breathing or respiratory					
	Tachypnoea	6342	1972 (31%)	2220 (26%)	25 570 (30%)
	Grunting	7687	637 (8%)	875 (10%)	7038 (8%)
	Indrawing	7780	1621 (21%)	782 (9%)	17 185 (20%)
	Crackles or crepitations	7863	1193 (15%)	699 (8%)	12 860 (15%)
	Respiratory signs	8161	3141 (39%)	401 (5%)	35 973 (42%)
Circulation					
	Capillary refill >2 s	6404	705 (11%)	2158 (25%)	9481 (11%)
	Temperature gradient	6270	655 (10%)	2292 (27%)	8921 (11%)
	Weak pulse volume	7381	946 (13%)	1181 (14%)	11 050 (13%)
	Circulatory signs	7697	1641 (21%)	865 (10%)	20 079 (23%)
Pallor		7954	1240 (16%)	608 (7%)	13 392 (16%)
Dehydration					
	Delayed skin pinch	7562	3569 (47%)	1000 (12%)	40 113 (47%)
	Sunken eyes	7511	3875 (52%)	1051 (12%)	42 843 (50%)
	Dehydrations signs	7889	4998 (63%)	673 (8%)	54 025 (63%)
	Dehydrations signs (severe)	7889	2446 (31%)	673 (8%)	28 931 (34%)
Disability or neurological					
	Convulsions	7836	856 (11%)	726 (9%)	9332 (11%)
	Inability to drink or breastfeed	7533	1864 (25%)	1029 (12%)	20 906 (25%)
	Impaired consciousness (AVPU<A)	7974	878 (11%)	588 (7%)	9453 (11%)
	Neurological signs	8118	2263 (28%)	444 (5%)	24 773 (29%)
Others					
	Malaria	8562	2771 (32%)	0	27 710 (32%)
	Death	8562	759 (9%)	0	7590 (9%)

Data are n (%) unless stated otherwise. AVPU=Alert, Voice, Pain, Unresponsive.

In 537 (6%) of 8562 patients, information on fluid prescribed was missing. Correct fluid (fluid type, volume, use of rate on infusion recommended for age of child) was prescribed in 3760 (44%) of 8025 participants. Among children with information on fluid prescribed, WHO plan B was correctly prescribed to 3398 (66%) of 5160 participants, WHO plan C to 331 (14%) of 2434 participants, and shock fluid prescriptions to 31 (7%) of 431 participants. 3569 (45%) of 8025 participants received at least some intravenous fluid whereas the rest of the participants received oral fluids only. Because intravenous fluids were more likely to be given to patients with more severe forms of dehydration, we included use of intravenous fluids in our analysis for risk factors as a proxy for disease severity. Antibacterials were prescribed in 5250 (61%) of 8562 participants, antimalarials in 2621 (32%) of 8252 participants, and any antimicrobial (antibacterial or antimalarial) in 6321 (74%) of 8562 participants. Electrolyte testing was generally unavailable across the hospitals.^9^

All variables included in the analysis had less than 27% of missing values (table 1), but a multilevel model using complete cases would have incorporated only 24% of cases (at least one missing variable). The population characteristics in the imputed data were similar to the characteristics before imputation (table 1).

**Figure 2:**
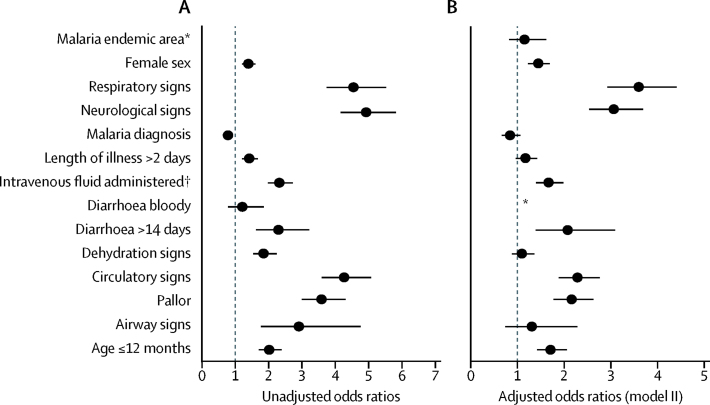
Risk factors for in-hospital mortality in children with diarrhoea and dehydration (A) Association of each covariable with in-hospital mortality in models using imputed data. (B) Association with in-hospital mortality after adjustment for all patient-level covariables. *Odds ratios not calculated. †Proxy measure for illness severity.

Analysis of the association of each covariable with mortality in models using imputed data suggested that female sex, age of 12 months or younger, length of illness of more than 2 days, diarrhoea duration of more than 14 days, abnormal airway signs, abnormal respiratory signs, abnormal circulatory signs, pallor, signs of dehydration, use of intravenous fluids (proxy for severity), and abnormal neurological signs were all significantly associated with in-hospital death across hospitals, whereas bloody diarrhoea and malaria parasitaemia were not (figure 2). Backward selection excluded bloody diarrhoea; as such, it was not included in the final multivariable model.

In model II, with adjustment of all patient-level covariables, age of 12 months or younger (adjusted-OR [AOR] 1·71, 95% CI 1·42–2·06), female sex (AOR 1·41, 1·19–1·66), diarrhoea duration of more than 14 days (2·10, 1·42–3·12), abnormal respiratory signs (3·62, 2·95–4·44), abnormal circulatory signs (2·29, 1·89–2·77), pallor (2·15, 1·76–2·62), use of intravenous fluids (1·68, 1·41–2·00), and abnormal neurological signs (3·07, 2·54–3·70) remained associated with in-hospital mortality within hospital clusters (figure 2). Notably, within this population, all of whom had diarrhoea and dehydration, individual signs of dehydration and presence of both sunken eyes and delayed skin pinch (severe dehydration) were not independently associated with in-hospital death (severe dehydration AOR 1·08, 0·87–1·35) within hospital clusters. However, when the same model was used in an analysis of the broader population of all patients with diarrhoea or dehydration (n=19 839; figure 1), signs of dehydration were independently associated with in-hospital death (AOR 1·22, 1·01–1·47; further data available on request). Length of illness of more than 2 days, abnormal airway signs, malaria diagnosis, and residence within a malaria endemic zone were not associated with death in the multivariable analysis (model II). Similar associations were seen when the multivariable model was done with the outcome as early in-hospital death, with the exception of diarrhoea duration of more than 14 days, which no longer showed a clear association with mortality (AOR 1·43, 0·87–2·34). When bloody diarrhoea was added to the final multivariable model, it was not associated with the outcome and the effect of the other covariates was not altered.

**Figure 3:**
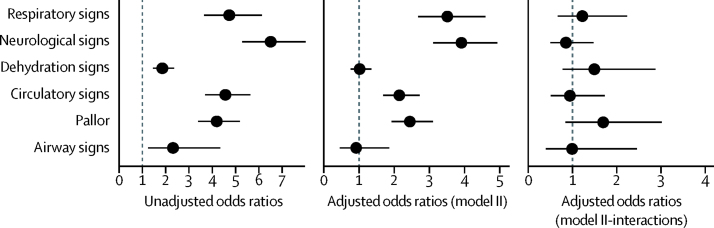
Risk factors and interaction with fluid management for early in-hospital mortality

In analyses restricted to the stratum for whom the fluid prescription was correct, the strength of association (ORs) with early in-hospital mortality was, in general, considerably lower than in the stratum in which fluid prescriptions were incorrect, even after adjustment for use of intravenous fluids (a proxy for severity). In fact, positive multiplicative interactions (OR>1) for association of incorrect fluid management with death in children with dehydration signs (OR 1·50, 95% CI 0·79–2·88), respiratory signs (OR 1·23, 0·68–2·24), and pallor (OR 1·70, 0·95–3·02) are evidence of interaction (figure 3). Furthermore, likely presence of additive interaction (relative excess odds due to interaction is not zero) point to the potential public health benefit of being able to correctly prescribe fluid regimens (table 2). The predicted probability of early in-hospital death was reduced by 6% when derived from model II with correct fluid prescription compared with when fluid prescription is wrong.

**Table 2:** Interactions of fluid management with risk factors for in-hospital early deaths

	**Multiplicative interaction**	**Fluid wrong and symptom present (OR10)**	**Fluid right and symptom present (OR11)**	**Fluid right and symptom absent (OR01)**	**RERI**	**Attributable proportion**
Pallor	1·70 (0·95 to 3·02)	2·16 (1·66 to 2·82)	1·07 (0·69 to 1·67)	0·29 (0·21 to 0·41)	−0·38 (−1·07 to 0·31)	−0·35 (−1·09 to 0·38)
Circulatory	0·95 (0·53 to 1·73)	1·92 (1·47 to 2·51)	0·64 (0·40 to 1·03)	0·35 (0·25 to 0·50)	−0·63 (−1·19 to −0·06)	−0·98 (−2·06 to 0·11)
Dehydration signs only	1·50 (0·79 to 2·88)	0·96 (0·70 to 1·33)	0·38 (0·26 to 0·55)	0·26 (0·15 to 0·45)	0·25 (−0·18 to 0·48)	0·40 (−0·53 to 1·33)
Neurological	0·86 (0·51 to 1·48)	3·57 (2·75 to 4·64)	1·15 (0·77 to 1·72)	0·37 (0·25 to 0·54)	−1·80 (−2·71 to −0·88)	−1·56 (−2·61 to −0·52)
Respiratory	1·23 (0·68 to 2·24)	3·18 (2·35 to 4·31)	1·17 (0·80 to 1·73)	0·30 (0·18 to 0·49)	−1·31 (−2·16 to −9·45)	−1·11 (−1·90 to −0·33)

Interaction exists if RERI is not zero (RERI=OR11 – OR10 – OR01 + 1). A negative value of RERI implies reduced risk due to interaction with fluid treatment. The attributable proportion is a measure of the proportion of the risk in the doubly exposed group that is due to the interaction itself. An interaction exists if the attributable proportion is not zero (attributable proportion=RERI/OR11). A negative value of the attributable proportion suggests that interaction reduces the risk of outcome (death). Multiplicative interaction=OR11/(OR10 ×   OR01). Presence of interaction on either additive or multiplicative scales indicates that both exposures have an effect on the outcome. The reference group was fluid wrong and symptom absent (OR00)=1·00. RERI=relative excess risk due to interaction.

## Discussion

A study^6^ using the Child Health and Nutrition Research Initiative's methodology recently identified risk factors for diarrhoeal deaths as a research priority. This study investigated clinical features associated with mortality and whether, for early deaths, associations are modified by prescribed fluid therapy in accordance with WHO and Kenyan guidance in children with both diarrhoea and dehydration. We used routinely collected data from 13 first-level referral hospitals in Kenya involved in a collaborative effort to improve availability of routine data on admitted children and the management of common conditions.^7^, ^8^, ^9^ We excluded children who had severe acute malnutrition, a known major risk factor for mortality with specific fluid management guidance,^7^, ^8^ and children younger than 30 days or older than 5 years because no standard rehydration guidelines exist for those ages. We did not have microbial cultures of stool or blood but malaria testing is done consistently by these hospitals in their own laboratories.^9^

In our study of 8562 children admitted with both diarrhoea and dehydration, we found that most (>80%) had at least one additional diagnosis (comorbidity). We also note that diarrhoea not accompanied by clinically diagnosed dehydration is a common symptom in a range of diseases. Among participants with diarrhoea and dehydration (representing 16% of all admissions and associated with 28% of all death), respiratory signs, circulatory impairment, pallor, neurological signs and symptoms, prolonged diarrhoea, age younger than 1 year, and female sex were associated with increased risk of in-hospital death. Our findings are consistent with studies that have shown impaired consciousness, convulsions,^18^ signs of pneumonia,^19^, ^20^ anaemia, weak pulse volume,^20^ longer duration of preadmission illness,^21^ and persistent diarrhoea^22^, ^23^, ^24^, ^25^ as risk factors for mortality in children with diarrhoea. Respiratory signs might in fact be indicative of pneumonia comorbidity. Female sex and younger age (<1 year), which were significantly associated with mortality in this study, have also been found to be risk factors in other studies. Our study did not investigate reasons for higher mortality in female participants than in male participants; some of the reasons suggested include social inequality and gender bias, but whether these factors are also important in Kenya is unclear.^26^

In our analysis of children with information on fluid prescribing, we found that correct fluid prescription was associated with reduced odds of death in participants with various clinical signs. Recommended fluid regimens are more complex for children who are more severely ill and might more often be wrongly prescribed. As well as adjusting for specific signs of severity, we also adjusted for the choice to use intravenous fluid therapy as a possible additional indication of the perceived disease severity. Overall, our findings suggest a potential public health benefit from ensuring or improving correct fluid prescription practices. In the exploratory analyses (data not shown), most incorrect fluid prescriptions in this study involved inadequate volumes or use of recommended volumes that were given over longer than recommended durations. Any efforts to improve correct fluid management will have to deal with the problem of comorbidity in children with diarrhoea and dehydration, something not covered in existing guidelines. The importance of tailoring fluid therapy to specific populations has been highlighted by the FEAST study. This large multicentre clinical trial of fluid treatment in African children with impaired perfusion and febrile non-diarrhoeal illnesses showed rapid fluid administration to be harmful in non-diarrhoeal cases.^27^ Special precaution was unlikely in the data presented because the data presented here represent prescription by front-line clinicians (mainly intern doctors or non-physician clinicians) who generally rely on guidelines and any decisions to deviate are made by senior clinicians much later after admission and are not reflected in this analysis.

Case fatality in the present study (9%) is similar to that found in a study^28^ involving two hospitals in western Kenya (9%); however, the patient population in the other study was different from ours because it included all children with diarrhoea and children with malnutrition. The study^28^ in western Kenya and the recent GEMS studies have shown that bacterial pathogens, including non-typhoidal *Salmonella, Cryptosporidium, Shigella*, and *E coli*, are important risk factors for death and severe disease and that rotavirus was not an important cause of in-hospital mortality.^5^ These findings have led to calls for studies on bacterial aetiology of diarrhoea and studies on antimicrobial resistance. Like the study in western Kenya, in our study, antibiotics were given to 60% of children with diarrhoea or dehydration, with comorbidities providing an indication for antibiotic use; however, most of the antibiotics used might not be effective against gut infections. Antibiotics are not routinely recommended and their use in the present study was a proxy for comorbidity.

The CIN has enabled improvements in documentation of the assessment, diagnosis, and treatment at admission of conditions since its inception and it uses a robust data capture system to try and optimise data quality.^7^, ^8^, ^9^ However, some data are missing and we employed imputation to maximise use of all available data. We do not have insight into whether the treatments prescribed at admission are accurately given—something that can be compromised by resource unavailability and staff shortages.^29^, ^30^ We also cannot be sure that clinicians are correct in their diagnosis and assessment or that they prescribe the right fluid regimens to the right patients. Data from such routine settings do not provide any insight on microbial aetiological diagnoses or on biochemical tests that might help assess severity of disease. Multiple imputation for missing data is based on an assumption that data are missing at random, which is difficult to ascertain. Finally, CIN does not collect data on all the care children receive across the period of their admission. Despite these limitations, this large dataset does offer a picture of routine admission characteristics and treatments across multiple sites and over a prolonged period of time, and findings might therefore be generalisable to similar African settings.

Children with diarrhoea and dehydration who are at most risk of in-hospital death are those with other signs of severe disease (comorbities) as opposed to those with uncomplicated dehydration. Importantly, prescription of recommended rehydration guidelines reduces risk of death in this group of children. However, correct fluid prescription might be an indicator of improved attention to the provision of good overall care, including other treatments such as antibiotics and nursing care, rather than fluid management alone. Strategies to optimise delivery of recommended guidance should be accompanied by studies on the management of dehydration in children with comorbidities, the vulnerability of female children, and the delivery of immediate care. Future research that investigates diarrhoea aetiology, that includes an enhanced range of diagnostic tests (including for comorbidities), and that effectively standardises the delivery of fluid therapy in accordance with guidelines, would help tease out more specific risk factors for poor outcome and could support newer management approaches that target more specific patient groups.

## Footnotes

* Abnormal airway signs include obstructed (stridor) or absent breathing, central cyanosis, or severe respiratory distress. Only stridor was included in the analysis as the other signs were rare while severe respiratory distress is not collected in the database.
